# Performance of complementary therapies in controlling inflammatory signs, symptoms, and complications after lower third molar extraction: a bibliometric analysis of the top 100 most-cited articles

**DOI:** 10.1590/acb410626

**Published:** 2026-02-06

**Authors:** Vinícius Lima de Almeida, Danilo Cassiano Ferraz, Giovanna Miranda Cabral, Arthur Henrique Gobbi, Walbert de Andrade Vieira, Lívia Bonjardim Lima, Rafael Rodrigues Lima, Sigmar de Mello Rode, Luiz Renato Paranhos

**Affiliations:** 1 Universidade Federal de Uberlândia – School of Dentistry – Post-Graduation Program in Dentistry – Uberlândia (MG) – Brazil.; 2 University of Pittsburgh – School of Dental Medicine – Pittsburgh (PA) – United States of America.; 3 Universidade Federal de Uberlândia – School of Dentistry – Graduation in Dentistry – Uberlândia (MG) – Brazil.; 4 Centro Universitário das Faculdades Associadas de Ensino – Department of Dentistry – São João da Boa Vista (SP) – Brazil.; 5 Universidade Federal de Uberlândia – School of Dentistry – Department of Oral, Maxillofacial, Traumatology, and Implantodontia Surgery – Uberlândia (MG) – Brazil.; 6 Universidade Federal do Pará – Institute of Biological Sciences – Laboratory of Functional and Structural Biology – Belém (PA) – Brazil.; 7 Universidade Estadual Paulista – Institute of Science and Technology – Department of Dental Materials and Prothesis – São José dos Campos (SP) – Brazil.; 8 Universidade Federal de Uberlândia – School of Dentistry – Department of Orthodontics – Uberlândia (MG) – Brazil.

**Keywords:** Pain, Trismus, Postoperative Period, Wound Healing

## Abstract

**Purpose:**

To conduct a bibliometric analysis of the 100 most-cited articles on the use of complementary therapies for controlling inflammatory signs, symptoms, and complications after lower third molar extraction.

**Methods:**

An electronic search was performed on the Web of Science Core Collection database. A graphical bibliometric network was created in Power BI software (Microsoft, Redmond, WA, United States of America). Spearman’s correlation test was used to assess the correlation among the number of citations, journal impact factor, and the year of publication.

**Results:**

The selected articles received 4,199 citations, covering the years 1983 to 2023. Most publications occurred in 2016. Article citations averaged 41.99. The most frequent Keywords Plus terms, considering more than five occurrences, were “removal,” “pain,” “surgery,” and “trismus.” The main keywords defined by the authors, considering more than five occurrences, were “pain,” “swelling,” and “trismus.” The Asian and European continents presented the highest number of publications and citations. Randomized clinical trial was the most prevalent study type. Regarding authors’ affiliations, the University of Turin stood out in the number of publications.

**Conclusion:**

The article with the highest number of citations was published in Serbia. Turkey and Italy accounted for the highest number of publications and citation density. Although most included studies were randomized clinical trials, improving comparison and decision-making requires methodological standardization.

## Introduction

Lower third molar extraction may be associated with inflammatory signs, symptoms, and postoperative complications, such as pain, edema, difficulty opening the mouth, alveolar osteitis, and infection^1^. These events may significantly compromise patient comfort and the quality of life in the days following surgery^2^. Postoperative consequences may concern factors such as the surgeon’s experience, surgical time, the positioning of said dental elements in the mandible, and the degree of individual inflammatory response^3^. Traditionally, managing these variables involves medications, which, although effective, might present undesirable side effects and contraindications^4^.

Developing complementary strategies to optimize tissue repair, aiming to restore tissue physiology and minimize postoperative consequences, is a constant challenge in contemporary dentistry^5^. Several therapies are employed for this purpose, each with its own advantages and limitations, including low-intensity laser therapy^6^, cryotherapy^7^, hyaluronic acid gel^8^, acupuncture^9^, photobiomodulation^10^, ozone therapy^11^, compression dressings^12^, herbal medicines^13^, and the administration of blood products^14^.

The scientific literature presents promising results regarding the effectiveness of these therapies, although there are controversies such as the lack of standardized protocols (variation in the measurement of pain and edema), limited rigor concerning clinical subgroups (smoking, diabetes and age), scarcity of medium- and long-term postoperative evaluations, and a lack of data on cost-effectiveness and patient acceptability^15^^,^ 16. Using these practices requires a critical and evidence-based overview to ensure a safe and effective incorporation into clinical practice^5^.

Thus, the bibliometric analysis provides a robust method for verifying research niches^17^. By mapping citation trends, highlighting influential studies, and identifying collaboration networks, bibliometric approaches offer considerable insights into the evolution and impact of scientific research^18^^,^ 19. To date, no bibliometric study with this theme has been performed.

Thus, the present study aimed to conduct a bibliometric analysis of the 100 most-cited articles on the use of complementary therapies for controlling inflammatory signs, symptoms, and complications after lower third molar extraction, providing comprehensive and up-to-date knowledge on mapping the state of the art, identifying scientific leaders and collaborations, evaluating the most studied therapies, and supporting the scientific foundation for conducting experimental studies and making clinical decisions.

## Methods

### Sources of information

This bibliometric review was conducted and reported in accordance with the Guidelines for Reporting Bibliometric Reviews of Biomedical Literature (BIBLIO). The Web of Science Core Collection (WoS-CC) was selected as the primary source of bibliometric data because it is one of the most comprehensive databases for citation analysis, indexing all article types, authors, institutional affiliations, and complete reference lists^20^. To ensure broad interdisciplinary coverage and the retrieval of complete metadata required for bibliometric analyses, all records were obtained exclusively from the WoS-CC. The data extraction was carried out on April 30, 2025.

### Search strategy

The search strategy was developed to capture publications related to lower third molars, signs and symptoms, postoperative complications, and complementary or adjunctive therapies used in their management. Three conceptual blocks were structured, each including synonyms and controlled vocabulary terms. Search descriptors were obtained from Medical Subject Headings (MeSH), Embase Subject Headings (Emtree), and Health Sciences Descriptors (DeCS). Boolean operators “AND” and “OR” were used to combine the terms, according to the syntax rules of the Web of Science database (Table 1).

**Table 1:** Database search strategy.

Database	Search Strategy (March 2025)
Primary Database	
Web of Science http://apps.webofknowledge.com/	#1 “Lower Third Molar” OR “Wisdom Teeth” OR “Wisdom Tooth” OR “Third Molar” OR «Mandibular Third Molar» #2 “Pain” OR “Swelling” OR “Trismus” OR “Alveolitis” OR “Alveolar Osteitis” OR “Dry Socket” OR “Socket” OR “Fibrinolytic Alveolitis” OR “Infection” OR “Cellulitis” OR “Abscess” OR “Recovery” OR “Healing” OR “Wound” OR “Outcomes” OR “Efficacy” OR “Morbidities” OR “Comparison” OR «Complications» OR «Edema» OR «Mouth Opening” #3 “PRF” OR “CGF” OR “PRP” OR “Platelet Rich Fibrin” OR “Platelet-Rich Fibrin” OR “IPRF” OR “Leukocyte and Platelet Rich Fibrin” OR “Concentrated Growth Factor” OR “Platelet-Rich Plasma” OR “Platelet Rich” OR “Platelet-Rich” OR “Hyaluronic Acid” OR “Cryotherapy” OR “Cold Therapy” Or “Phytotherapy” OR “Laser Therapy” OR “Low-Level Light Therapy” OR “Drain” OR “Brief Hypnotic Induction” OR “Green Tea” OR “Propolis” OR «Suture Techniques» OR “Piezosurgery” OR «Acupuncture» OR «Herbal Medicine» OR «Curcumin» OR «Bromelain» OR “Arnica» OR «Photobiomodulation» OR «LLLT» OR «PBM» OR «Autologous Conditioned Plasma» OR «A-PRF» OR “T-PRF» OR “Cryotherapy» OR «Ozone” OR “Therapy” OR «Ozonated Water” OR “Minimal» or «Transcutaneous Electrical Nerve Stimulation” OR “TENS» OR “Ultrasound» OR «Kinesio Taping” OR “PRGF» OR «Plasma Rich in Growth Factors” OR «Collagen Membrane” OR «Collagen Resorbable Membrane” OR “Chitosan» OR «Pulsed Electromagnetic Field” OR «Probiotic»
	**#1 AND #2 AND #3**

Source: Elaborated by the authors.

The search was performed using the “Classic Search” interface (with the option “Try the new Web of Science” unchecked), under the “Documents” tab. Each search string (detailed in Table 1) was entered in the “Topic” field, which includes the title, abstract, authors’ keywords, and Keywords Plus®. After executing the search, the resulting records were sorted in descending order by the number of citations, allowing the identification of the most influential articles within the dataset.

### Time period

The database search included all articles published up to April 2025, with no restriction on the starting date, in order to provide a complete historical overview of the scientific output on the topic.

### Eligibility criteria

The study included the 100 most-cited articles that investigated the performance of complementary therapies in controlling inflammatory signs, symptoms, and complications after lower third molar extraction. There was no restriction on the year or language of publication.

### Selection process

Since only one database was used for the search, there was no need to check for duplicate documents. The study selection was therefore conducted in a single phase by a pair of calibrated reviewers. For articles published in languages other than Portuguese, English, and Spanish, a translation tool was used for the initial analysis of their titles and abstracts. The reviewers analyzed the titles and abstracts of all records identified in the search to determine final eligibility, with inclusion and exclusion decisions made jointly and by consensus. The study protocol stipulated that, if the analysis of a title and abstract was insufficient for a consensual decision, the full text would be read to solve the disagreement. If disagreement persisted even after full-text reading, the final decision would be made by a third reviewer.

### Data extraction from the selected studies

From the final list of eligible records, the 100 most-cited articles were selected for an in-depth analysis. This selection was based on citation data provided by the Web of Science Core Collection in April 2025.

For this subset of articles, two reviewers extracted the data by consensus. The following information was collected: year of publication, number of citations, position in the list of citations, citation density, the number of general citations, title, authors, institution, country, journal, impact factor according to the Journal Citation Reports (Clarivate Analytics), keywords, study design, open access policies, and other topics of interest. Study designs were categorized into systematic reviews, systematic reviews with meta-analyses, umbrella reviews, narrative reviews, literature reviews, clinical practice guidelines, prospective articles, clinical trials, observational studies (cohort, cross-sectional, and case-control), and *in-vitro*/animal studies. The collected data from countries and institutions were based on information from the corresponding authors. International collaboration considered the institution of origin of each author and co-author. Two evaluators (VLA and DCF) analyzed the data independently to minimize error. The topic of interest evaluated in this bibliometric analysis was the performance of complementary therapies in controlling inflammatory signs, symptoms, and complications after lower third molar extraction.

### Data processing and standardization

After extraction, a data processing and standardization procedure was carried out to ensure the accuracy and consistency of the bibliometric analysis. This process included the unification of variations in authors’ names, the standardization of country and institution names, and the grouping of synonymous keywords or those with minor spelling variations. For records with incomplete data, such as missing authors’ affiliation or country information, a manual search was performed in external sources to complement the data. When the information could not be found, it was classified as “undefined.” The protocol established that potential citation outliers would not be removed, as they represent influential milestones and constitute valid data points for the bibliometric analysis.

### Quality assessment

A formal assessment of the methodological quality or risk of bias of the included articles was not performed. Such a procedure does not apply to the scope of this study, whose primary selection criterion was the impact of the articles in the literature, measured by the number of citations.

### Data synthesis and analysis

#### Bibliometric analysis

A graphical bibliometric network was created in Power BI software (Microsoft, Redmond, WA, United States of America) to analyze publication trends, study design, topic selection, geographical distribution, international collaborations, authorship analysis, institutional affiliations, and keyword analysis. Full counting was adopted, assigning each co-author one unit of credit per publication. The data related to connections between authorship and keywords were clustered. Terms associated with clusters and larger fonts had higher occurrences, while terms associated with clusters or smaller fonts had lower occurrences. Connection lines were drawn between clusters to indicate relationships.

#### Statistical analysis

Spearman’s correlation test was used to assess the correlation among the number of citations, journal impact factor, and year of publication. Spearman’s correlation coefficient (rho) may be weak (rho > 0.30), moderate (rho > 0.50), high (rho > 0.70), or very high (rho > 0.9). All analyses were performed in R software (version 4.5 for Windows) at a 5% significance level.

## Results

### Descriptive findings

The search strategy in the WoS-CC database yielded 888 studies, which were classified by the number of citations. After screening, the 100 most-cited articles were selected^21-120^. Kaziro^21^ authored the first article to analyze the performance of complementary therapies in controlling inflammatory signs, symptoms, and complications after lower third molar extraction. It was published in the *British Journal of Oral and Maxillofacial Surgery* and received 54 citations. The most recent article, by Elayah et al.^22^, was published in *Frontiers in Endocrinology* and received 21 citations. A very weak positive correlation was found between journal impact factor and the number of citations (rho = 0.249, *p* = 0.01).

**Figure 1:**
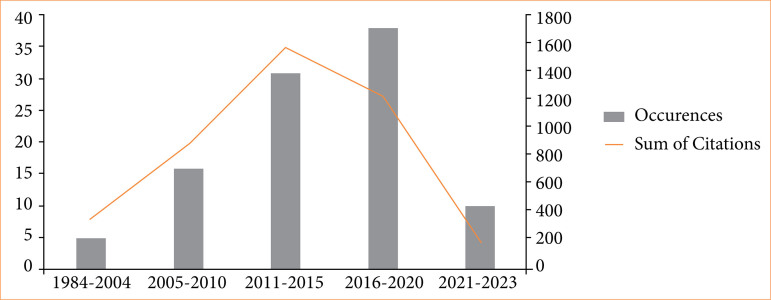
Trend in publications and citations over time. The highest number of publications occurred in 2016 (n = 12).

Source: Elaborated by the authors.

**Figure 2:**
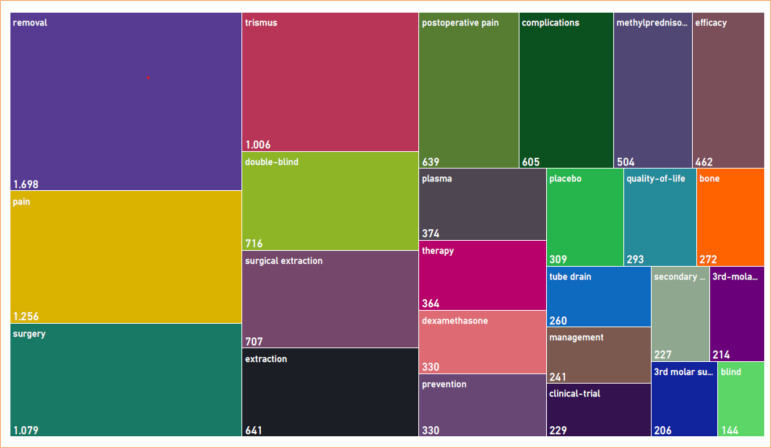
Tree map of Keywords Plus, which are the index terms automatically generated from the titles of cited articles in the Web of Science Core Collection database. Larger boxes indicate a higher sum of citations. The most frequent Keywords Plus terms, considering more than five occurrences, were “removal”, “pain”, “surgery”, and “trismus.”

Source: Elaborated by the authors.

**Figure 3:**
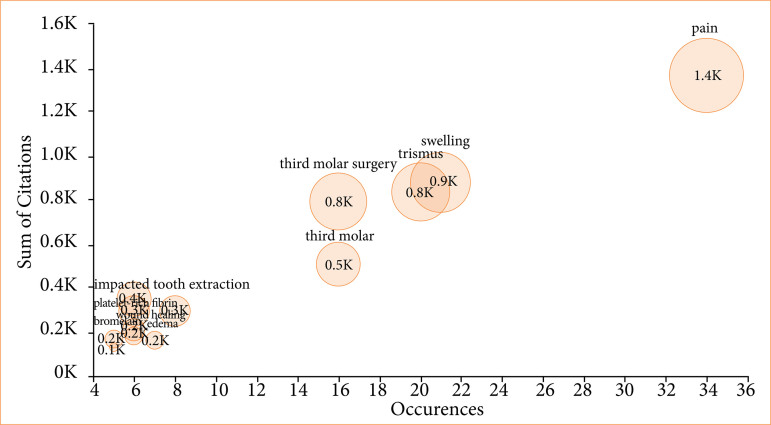
Keywords defined by the authors, considering more than five occurrences. The main keywords defined by the authors were “pain”, “swelling”, and “trismus.”

Source: Elaborated by the authors.

The selected articles received 4,199 citations, covering the years 1984 to 2023. The highest number of publications occurred in 2016 (n = 12), as shown in Fig. 1, which also presents citation trends. There was a moderate negative correlation between the year of publication and the number of citations (rho = -0.597, *p* < 0.001). Article citations ranged from 10 to 125, with an average of 41.99 citations per article. The most cited article, a randomized clinical trial published in the *International Journal of Oral and Maxillofacial Surgery* in 2007 by Markovic and Todorovic, had 125 citations^23^. The most frequent Keywords Plus terms, considering more than five occurrences, automatically generated by WoS-CC were “removal” (1,698 citations), “pain” (1,256 citations), “surgery” (1,079 citations), and “trismus” (1,006 citations), as seen in Fig. 2. The main keywords defined by the authors, considering more than five occurrences, were “pain” (1,400 citations), “swelling” (900 citations), and “trismus” (800 citations), as shown in Fig. 3.

### Schematic map and trend

**Figure 4:**
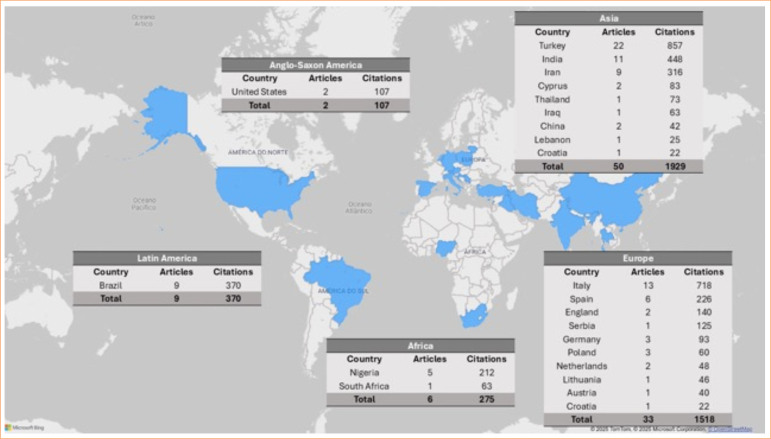
Distribution of publications and citations of the 100 most-cited articles across continents, based on corresponding authors. Regarding geographical distribution, Asia had the highest number of publications and citations, followed by Europe, Latin America, Africa, and Anglo-Saxon America.

Source: Elaborated by the authors.

**Figure 5:**
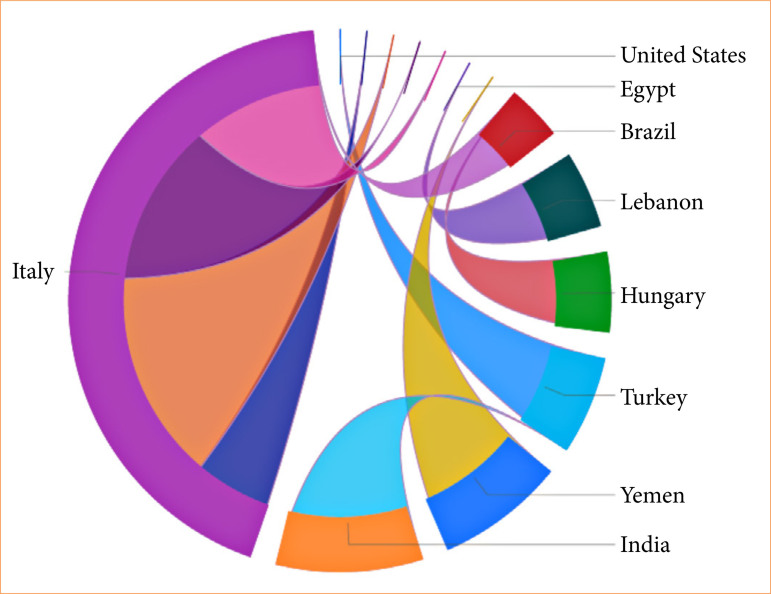
International collaborations are visualized through a chord diagram, reflecting affiliations of all contributing authors. The colors of the chords represent the destination of each collaboration. Among the 100 most-cited articles, only nine countries collaborated, with Italy having the highest number of international collaborations and consequently more citations.

Source: Elaborated by the authors.

Regarding geographical distribution, Asia had the highest number of publications and citations (50% of articles, 1,929 citations), followed by Europe (33 articles, 1,518 citations), Latin America (nine articles, 370 publications), Africa (six articles, 275 publications), and Anglo-Saxon America (two articles, 107 citations). Turkey stood out with 22% of publications and 857 citations. Italy (13%, 718 citations), India (11%, 448 citations), Brazil (9%, 370 citations), Iran (9%, 316 citations), Spain (6%, 226 citations), and Nigeria (5%, 212 citations) individually exceeded 200 citations, as seen in Fig. 4. Figure 5 illustrates the international collaboration network of countries. Among the 100 most-cited articles, only nine countries collaborated, with Italy having the highest number of international collaborations and consequently more citations (six articles, 237 citations).

**Figure 6:**
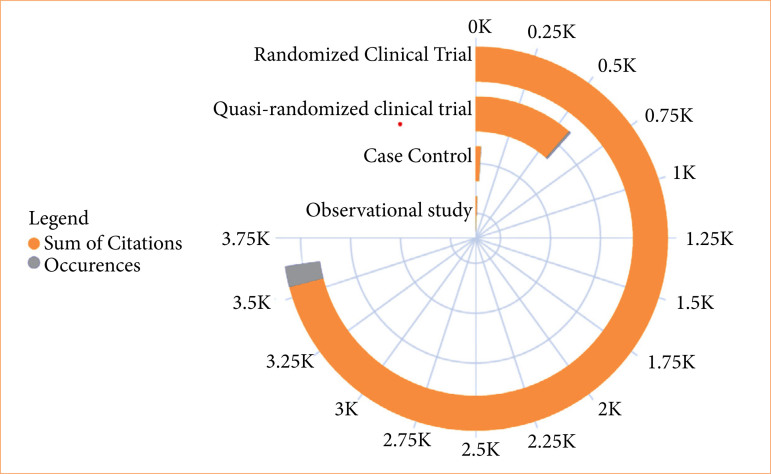
Prevalence of articles and citations across various study designs within the top 100 studies. Only one article was identified for case-control and observational study designs.

Source: Elaborated by the authors.

The randomized clinical trial was the most prevalent study type (87% of selected articles) and obtained the highest number of citations (3,548 citations), followed by quasi-randomized clinical trials (11% of articles, 571 citations). Only one article was identified for case-control (48 citations) and observational (32 citations) study designs, as shown in Fig. 6.

**Figure 7:**
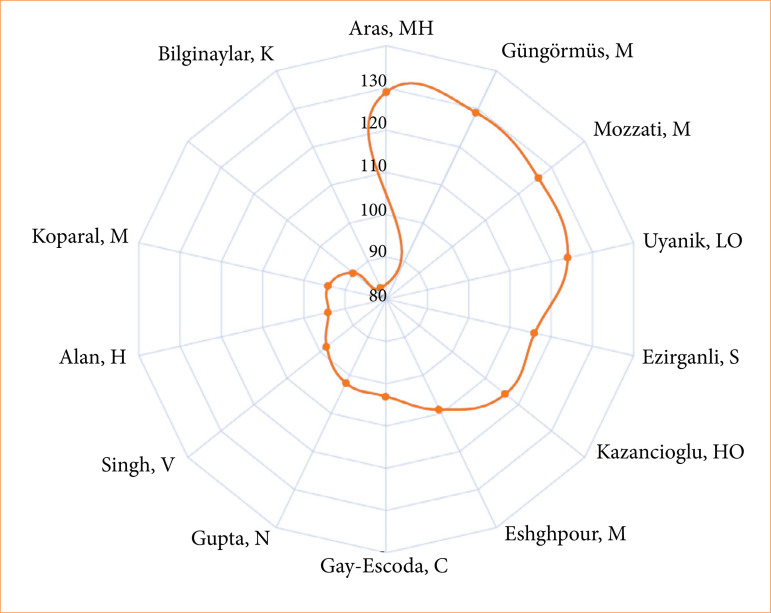
Radar chart showing authors with two or more occurrences and 80 or more occurrences: Aras MH and Gungormus M (129 citations), Mozzati M (126 citations), Uyanik LO (124 citations), and Ezirganli S (116 citations).

Source: Elaborated by the authors.

Among the 100 most-cited articles, only two were published by a single author^21^^,^ 24. The radar plot (Fig. 7) shows the authors with two or more occurrences and 80 or more citations: Aras MH and Gungormus M (129 citations), Mozzati M (126 citations), Uyanik LO (124 citations), and Ezirganli S (116 citations).

**Figure 8:**
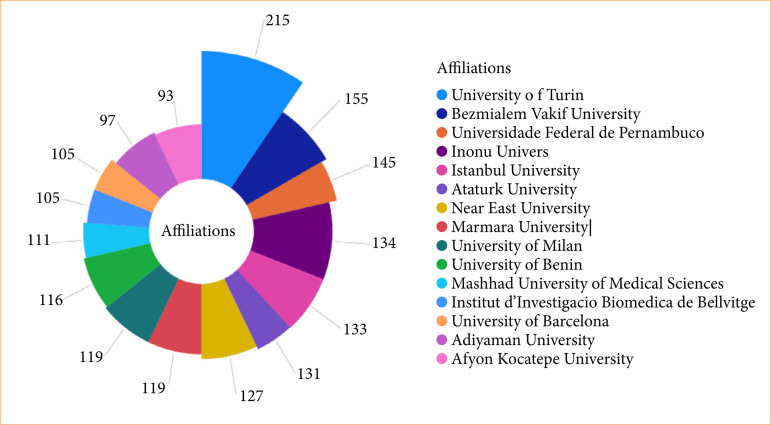
Aster diagram demonstrating author affiliations, considering those with two or more occurrences and 80 or more citations. The University of Turin stood out with four articles and 211 citations, followed by Bezmialem Vakif University, Universidade Federal de Pernambuco, Inonu University, and Istanbul University.

Source: Elaborated by the authors.

Regarding authors’ affiliations, the Aster plot in Fig. 8 shows the five main institutions, considering only those with two or more occurrences and 80 or more citations. The University of Turin stood out with four articles and 211 citations, followed by Bezmialem Vakif University (three articles, 152 citations), Universidade Federal de Pernambuco (two articles, 143 citations), Inonu University (four articles, 130 citations), and Istanbul University (three articles, 130 citations).

## Discussion

Developing complementary therapies for controlling inflammatory signs, symptoms, and complications after lower third molar extraction is a trend in contemporary dentistry^5^. This bibliometric analysis is the first study to analyze the top 100 most-cited articles in the WoS-CC database.

The WoS-CC database was used exclusively for this bibliometric study due to its standardized citation data and methodological consistency. The use of additional databases, such as Scopus or PubMed, although potentially expanding coverage, would introduce metadata heterogeneity, require greater harmonization efforts, and could potentially lead to methodological bias^20^^,^ 121. Moreover, since the present analysis focuses on co‐citation networks and thematic evolution over time, the structured citation network of WoS meets the study’s requirements.

In this study, the article that received the most citations was by Markovic and Todorovic^23^, with 125 citations, while the study by Momeni et al.^120^ received only 10 citations. Factors such as methodological quality, journal impact factor, and collaboration research network may influence citation metrics^122^. However, the article that received the fewest citations was published in *BMS Oral Health*, whose impact factor is 3.1, which is higher than the impact factor of the study with the most citations, published in the *International Journal of Oral and Maxillofacial Surgery*. This difference may be linked to factors that influence citation metrics, such as the novelty of the researched theme and the time of article publication^123^. It is worth noting that Spearman’s test presented a very weak positive correlation between journal impact factor and the number of citations (rho = 0.249, *p* = 0.01).

Randomized clinical trial (RCT) was the most frequent study type, representing 87% of the selected articles. This study design randomly selects patients to receive the intervention or control, ensuring the balance of potential known and unknown confounding factors at the time of randomization^124^. Moreover, the RCT may provide strong evidence for research evaluating the effectiveness of a clinical intervention, aiding evidence-based decision-making^125^. However, although most selected studies were RCTs, the comparison of interventions presents methodological flaws, as some studies lack sample calculation, parallel and split-mouth designs, and heterogeneous positioning of third molars in the mandible. These points are outside the protocol suggested by the Consolidated Standards of Reporting Trials (CONSORT) and may influence the interpretation, inference of results, and decision-making^126^.

Asia and Europe have the highest number of articles among the 100 most cited on complementary therapies for controlling inflammatory signs, symptoms, and complications after lower third molar extraction. This data contrasts with Huang et al.^127^, whose bibliometric analysis showed that Brazil contributed to the highest number of articles among the top 100 most cited, including research on pain control and drug use after lower third molar extraction. This difference may be attributed to the larger number of therapies analyzed and the broader scope of research lines and groups included in this study. Although Brazil does not rank in a prominent position regarding the number of scientific publications among the top 100 most-cited articles in this study, the Universidade Federal de Pernambuco have two articles and 143 citations among the five most cited higher education institutions. The four other most cited institutions are in Asia and Europe, corroborating the continents that contributed to the most published articles on this subject.

Turkey and India are among the countries with the most scientific contributions in the top 100 most-cited articles, and they stand out in the research on complementary therapies after lower third molar extraction, mainly addressing the application of laser therapy and blood concentrates. These countries have consolidated their presence in the global scientific research landscape through significant advances in production and infrastructure. India, which became the third country to publish the most scientific articles in 2022, with 177,291 articles, registered a 19% growth compared to the previous year^128^. Turkey has significantly expanded its presence in international scientific research, reflected by several bibliometric indicators. From April 1, 2024, to March 31, 2025, the country ranked 34th in the global ranking of scientific production by the Nature Index, and fourth in the Western Asia region, with emphasis on physical, health, biological, land, and environmental sciences^129^.

Keywords are critical for the effectiveness of a literature search strategy, as they condense longer sentences or phrases and expand the ability to track articles^130^. In the present study, the main keywords defined by the authors were “pain,” “swelling,” and “trismus.” Notably, these terms correspond to the most frequent inflammatory signs and symptoms after lower third molar extraction, which justifies the emphasis of such terms as the main keywords referring to the top 100 most-cited articles. Although pain, edema, and difficulty opening the mouth are common after this surgical procedure, the intensity of these inflammatory signs and symptoms might be strongly related to the degree of surgical difficulty, individual tissue repair response, and the surgeon’s experience^100^^,^ 131.

Although it is relevant as an analysis method for mapping existing research and providing knowledge of the geographic distribution and influence of each continent regarding their scientific contribution, this bibliometric analysis has some limitations. Robust studies on the subject exist, but they may not have been included due to the recent date of publication, which consequently yields few citations. The WoS-CC database is among the most reliable and widely used citation databases for bibliometric studies^132^. However, as it was the only database used, other robust studies with a high number of citations may not have been retrieved. Furthermore, the lack of methodological standardization and certain limitations in the primary studies are evident, such as the absence of sample size calculation, heterogeneous therapeutic protocols, and direct comparisons among patients with lower third molars presenting different degrees of surgical complexity. To minimize the risk of bias and ensure reproducibility and effective clinical application of the results, it is suggested that randomized clinical trials be conducted with methodological standardization.

## Conclusion

This bibliometric analysis identified the 100 most-cited articles on the performance of complementary therapies in controlling inflammatory signs, symptoms, and complications after lower third molar extraction. The article with the highest number of citations was published in Serbia. The Asian and European continents presented the highest number of studies, with Turkey and Italy accounting for the most publications and density of citations. Although most included studies were randomized clinical trials, improving comparison and clinical evidence-based decision-making requires methodological standardization. Global methodological standardization is required to ensure the safe and effective clinical incorporation and reproducibility of complementary therapies for the control of signs, symptoms, and complications following lower third molar extraction.

## Data Availability

The data will be available upon request.
